# Anatase-cellulose acetate for reinforced desalination membrane with antibacterial properties

**DOI:** 10.1186/s13065-023-01013-1

**Published:** 2023-09-12

**Authors:** Ahmed S. Abdel-Fatah, Hebat-Allah S. Tohamy, Sayed I. Ahmed, Mohamed A. Youssef, Mohamed R. Mabrouk, Samir Kamel, Farag A. Samhan, Ayman El-Gendi

**Affiliations:** 1 Water Quality Audit Department, Egyptian Water and Wastewater Regulatory Agency, New Cairo City, Egypt; 2 Cellulose and Paper Department, National Research Centre, Cairo, 12622 Egypt; 3 Faculty of Engineering, Public Works Dept., Ain Shams University, Abbasseya, Cairo, 11535 Egypt; 4 Department of Chemistry, Faculty of Science, Helwan University, Cairo, Egypt; 5 Environmental and Climate Change Research Institute, National Research Centre, Cairo, 12622 Egypt; 6 Chemical Engineering and Pilot Plant Department, Engineering Research and Renewable Energy Institute, National Research Centre, Cairo, 12622 Egypt; 7 Canal High Institute of Engineering and Technology, Ministry of High Education, Suez, Egypt

**Keywords:** Cellulose acetate, Anatase, Membranes, Desalination, Antibacterial, Permeability, Mechanical properties

## Abstract

This study aimed to prepare antifouling and highly mechanical strengthening membranes for brackish and underground water desalination. It was designed from cellulose acetate (CA) loaded anatase. Anatase was prepared from tetra-iso-propylorthotitanate and carboxymethyl cellulose. Different concentrations of anatase (0.2, 0.3, 0.5, 0.6, 0.7, and 0.8)% were loaded onto CA during the inversion phase preparation of the membranes. The prepared membranes were characterized using Fourier Transform Infrared spectroscopy (FTIR), X-ray diffraction (XRD), thermogravimetric analysis (TGA), scanning electron microscopy (SEM & EDX), mechanical properties, swelling ratio, porosity determination, and ion release. The analysis confirmed the formation of anatase on the surface and inside the macro-voids of the membrane. Furthermore, anatase loading improved the CA membrane’s mechanical properties and decreased its swelling and porosity rate. Also, CA-loaded anatase membranes displayed a significant antibacterial potential against Gram-positive and Gram-negative bacteria. The results showed that the salt rejection of the CA/anatase films as-prepared varies considerably with the addition of nanomaterial, rising from 46%:92% with the prepared membranes under the 10-bar operation condition and 5 g/L NaCl input concentration. It can be concluded that the prepared CA-loaded anatase membranes have high mechanical properties that are safe, economical, available, and can stop membrane biofouling.

## Introduction

Water scarcity is caused by excessive water use in many parts of the world, and more than two billion people are expected to suffer from it. Due to water scarcity, Egypt will be one of the world’s most dangerous countries in the next ten years [1]. In 2015, the human water share in Egypt was 616 m^3^/capita/year, which gradually decline to 392 and 342 m^3^ in 2037 and 2050, respectively, and according to the United Nations, this is less than the water-poverty threshold of 1000 m^3^/year [2]. Water scarcity complicates the life cycle and jeopardizes the world’s population. Egypt’s rising water demand is primarily due to rapid population development, water pollution, and limited freshwater sources (Nile River, rains, underground wells, lakes, and streams) [3]. Egypt’s population expansion is one of the most serious dangers to the country’s agricultural and industrial future, as well as its water self-sufficiency [4]. It is expected to reach 120 million by 2030 [5]. In Egypt, surface water is the primary source of municipal and industrial activity, while groundwater and rainwater play a minor role in water resources [6]. According to climate change forecasts, the Middle East will see a 5–25% decline in annual precipitation, as well as an increase in sea level rise, and thus the hydraulic effect of groundwater will lead to increased seawater intrusion in many coastal aquifers [7]. Siwa Oasis has recently suffered from salinization resulting from seawater intrusion, and Many places in Egypt need desalination, such as Marsa Matruh, Al-Nagila, Barani, Salloum, Al-Dabaa, Madinaty, Central Sinai, Al-Fishah, Al-Arish, Rafah, Burj Al-Arab, Al-Nubaria and many other places [8]. Also, the Grand Ethiopian Renaissance Dam will reduce the water flow into the Nile River [3]. The synthesis of different metal oxide nanoparticles (NPs) by eco-friendly methods is a promising alternative to conventional chemical methods [9]. Polymers are organic molecules with many exceptional properties, including high mechanical strength, flexibility, chemical stability, and vast surface areas. These qualities allowed both organic and inorganic compounds to be hosted as polymers. As a result, we may produce different molecules with particular properties for water desalination and purification [10]. Cellulose is a biodegradable and cheap polymer on earth that can be recycled from agricultural wastes to make films and membranes. The current biodegradable properties of cellulose limit its practical applications [11, 12]. In this study, we will incorporate anastase between cellulose acetate (CA) membranes to reinforce it.

Several methods, such as creating drains, reusing treated wastewater, desalinating seawater, and extracting water from the air, can address future concerns [13].

The importance of the desalination process cannot be emphasized. The total global production of desalinated water has increased from approximately 5 million m^3^/day in 1980 to 20 million m^3^/day in 2000, rising to 90 million m^3^/day in 2020. The market for desalinated water has grown, and this trend is expected to continue in the coming decades [14]. Desalination is a strategic option as an alternative and unconventional water supply for Egypt [15, 16]. Egypt has enormous salty water reserves (long sea beaches, saltwater lagoons, brackish groundwater available from several aquifers, and extensive drainage networks). Cellulose acetate (CA) is a biodegradable and plentiful material that is good for the environment. Its strong biocompatibility, non-toxicity, great hydration, high flux, and facile film formation make it widely utilized in the separation, desalination, and treatment of various water bodies [17, 18]. Long-term research has concentrated on improving CA membrane filtration performance and mechanical properties [19].

On the other hand, metal oxide NPs have reportedly been used in membrane applications, such as Al_2_O_3_ [20], SiO_2_ [21], Ag [22], and Cu & Cu/Ag [23]. Because some of these metal oxide NPs are relatively expensive, efforts have been concentrated on selecting a lower-cost metal oxide. Among these oxides, Titanium oxide (TiO2) is a popular low-cost metal [24]. It has attracted more attention due to its suitability for applications like photocatalysis for self-cleaning surfaces, photoelectronic activity, and water purification, particularly membrane filtration [25, 26]. Under UV or visible light irradiation, phenols, alcohols, carboxylic acids, and dye can be photodegraded by TiO_2_NPs. Also, adding TiO_2_NPs to cellulose improved the mechanical properties of the cellulosic membrane [27]. Our analyses found that adding TiO_2_ to membranes can boost permeability while reducing or eliminating the possibility of microbiological fouling. TiO_2_ also has the potential to be used as a water disinfectant due to its ability to kill microorganisms. This research aims to identify an antifouling membrane that will be deployed in Egyptian villages and small communities to desalinate brackish and/or groundwater. The addition of anatase to cellulose acetate polymer represented the novelty element in this research. It improved the membrane properties for water treatment such as, anti-biofouling and mechanical properties. To the best of our knowledge, no published articles have been issued using anatase for this purpose, as it is novel and innovative in terms of green technology for Preparing Antifouling Desalination Membranes.

## Materials and methods

### Materials

The bleached cellulose bagasse pulp was delivered from Quena Company of Paper Industry, Egypt. The chemical position of the prepared pulp was cellulose content (96%), hemicellulose (3%), and very low lignin content. Cellulose acetate (hydrophilic) (Fluke biochemical CO, Switzerland) (Mwt: 37,000 g/mol, CAS number: 9004-35-7, purity = 40% acetyl groups), acetone(hydrophobic) (LOBA CHEMIE CO, India) (Mwt:58.8 g/mol, CAS number: 67-64-1, purity = 99.8%), ethanol (hydrophilic) (Merck) (Mwt: 46.07 g/mol, CAS number: 64-17-5, purity < 99%), tetra-iso-propylorthotitanate (hydrophobic) (Merck Schuchardt OHG CO, Germany) (Mwt: 284.22 g/mol, CAS number: 546-68-9, purity ≥ 98%), isopropanol (hydrophilic) (Merck) (Mwt: 60.1 g/mol, CAS number: 67-63-0, purity ≥ 99.9%), sodium hydroxide (NaOH) (hydrophilic) (Merck) (Mwt: 39.9 g/mol, CAS number: 1310-73-2, purity ≥ 98%), monochloroacetic acid (MCA), tryptone soya agar (India) (Concentration 40 gm/lit, CAS number: 0-0-0, purity = 95%).

### Methods

#### Preparation of carboxymethyl cellulose (CMC)

CMC was prepared by dispersing 4 g of bleached cellulose bagasse pulp in 75 ml isopropanol followed by addition of 7.5 ml NaOH (30%), and mixed at room temperature. After 30 min, 3.0 g of monochloroacetic acid (MCA) was added and stirred at 60 °C for 2.5 h, then washed with 70% ethanol [28].

#### Preparation of anatase

0.1 ml of tetra-iso-propylorthotitanate (C_12_H_28_O_4_Ti) was added to an aqueous CMC solution (5.0 g/80 ml H_2_O) and stirred for 5 min. The powder was calcinated at 500 °C for 2 h [25].

#### Preparation of membranes

**Fig. 1:**
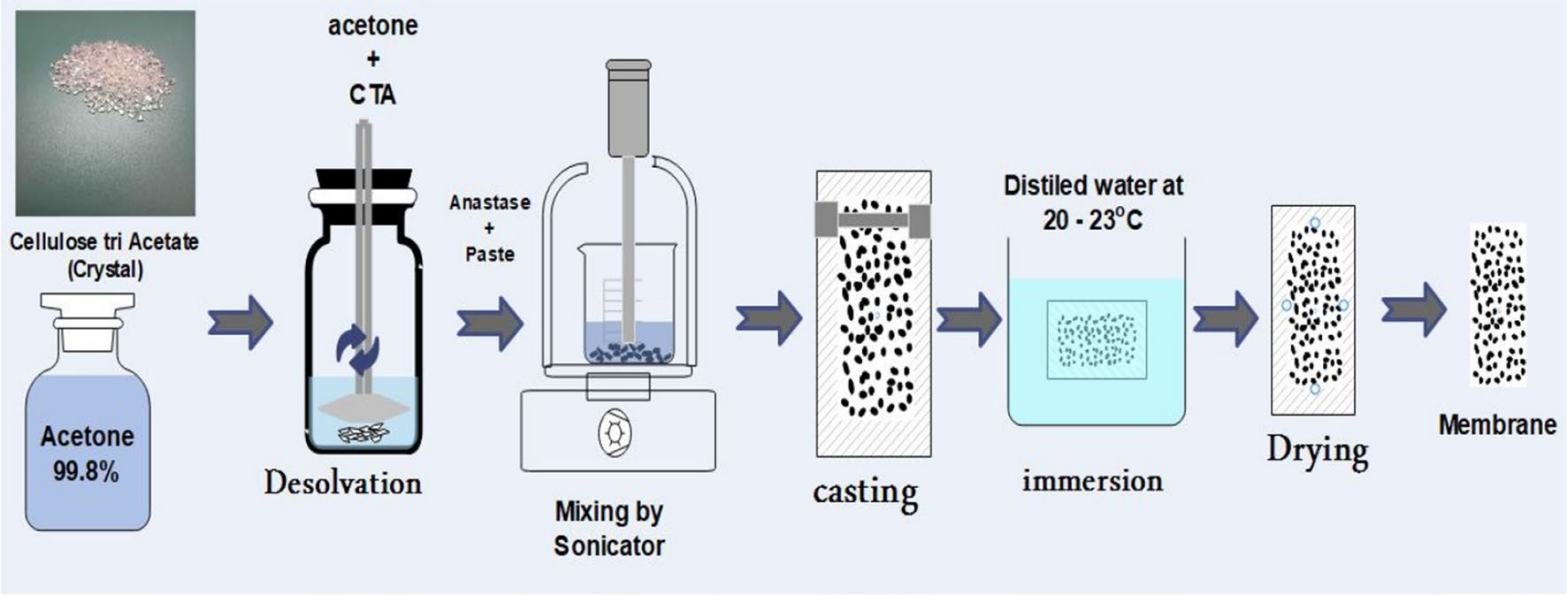
Steps of membrane preparation

Membranes were prepared by a Loeb–Sourirjan (L–S) wet phase inversion process. Different weights of anatase (0.0, 0.2, 0.3, 0.5, 0.6, 0.7, and 0.8 g)% were added, respectively, to (14.7, 14.5, 14.4, 14.2, 14.1, 14.0, and 13.9 g) of CA dissolved in 85.3 ml acetone and sonicated for 1 h. After evacuating the gasses, the solution was poured on plates in suitable amounts, and after being exposed to air for 10 s, the glass plate was submerged in a water bath at room temperature for 1 h. After coagulation and complete washing, membranes were kept for characterization and coded as; M0, M1, M2, M3, M4, M5, and M6 related to anatase weights, respectively. Figure 1 shows the main steps of membrane preparation.

#### Characterizations

**Fig. 2:**
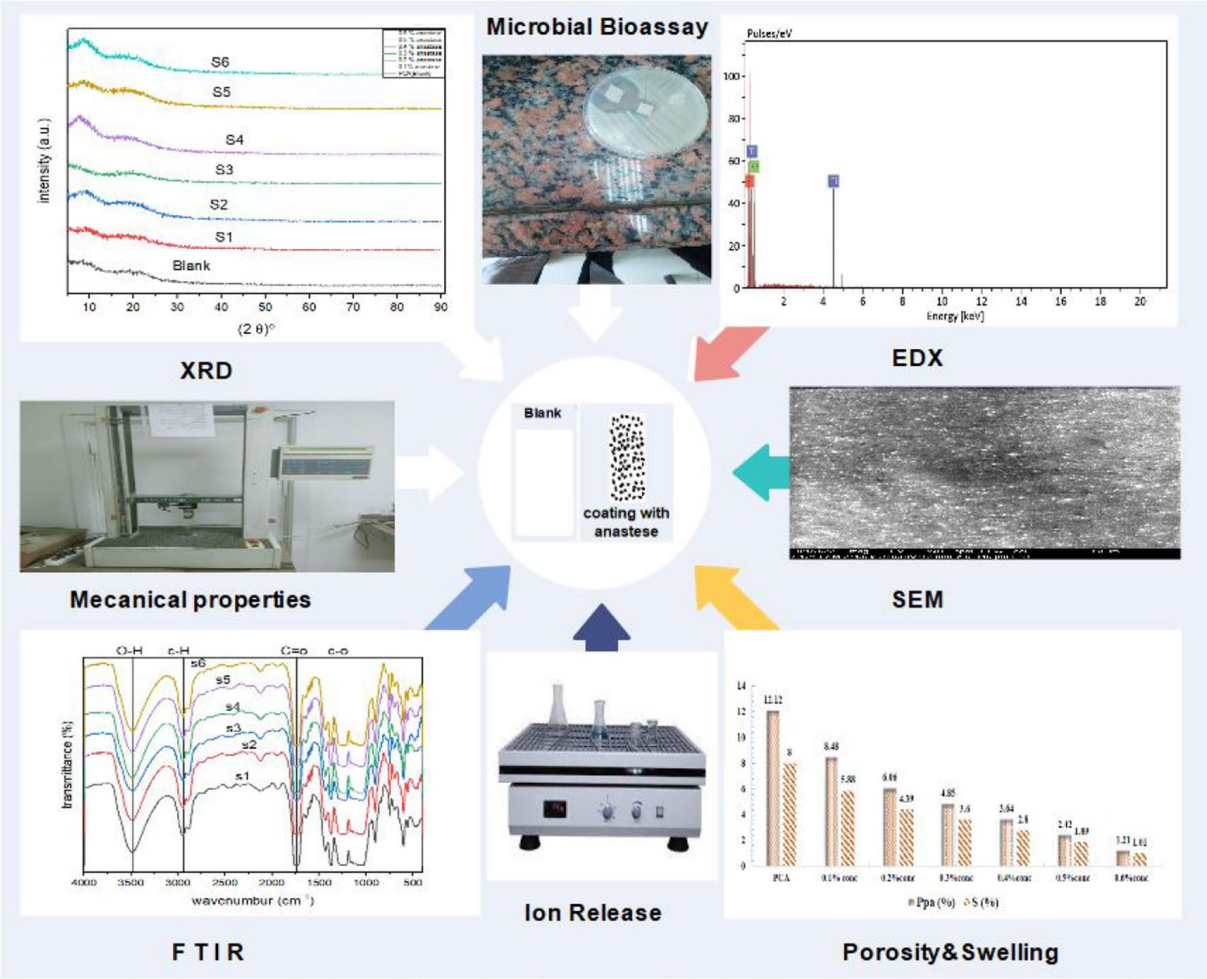
Graphical abstract of membrane characterization

After preparing the membrane, it was characterized using FTIR, XRD, TGA, SEM & EDX, mechanical properties, swelling ratio, porosity determination, and ion release (Fig. 2).

#### Fourier transform infrared spectroscopy (FTIR)

FTIR spectra were recorded for produced membranes by FTIR spectrophotometer (Prestige-21, Shimadzu (Japan). The air background was taken before each sample scan. The frequency range was from 500 to 4000 cm^−1^ having a resolution of 4.0 cm^−1^ with an average of 200 scans per spectrum.

#### X-ray diffraction

X-ray diffraction patterns of membranes were obtained with XRD (Bruker model D2 PHASER diffractometer) using Nickel-filtered CuKα radiation (30 kV, 10 mA). The scanned rate was 0.02°/min ranging from 5 to 80° (2θ) with a wavelength of 0.154 nm.

$${\text{Crystalinity index}}\left( {{\text{Cr}}.{\text{I}}} \right)\% { } = \frac{{\text{Total area of crystalline peaks }}}{{\text{Total area of crystalline and amorphous peaks}}} \times 100$$

#### Thermogravimetric analysis (TGA)

Using a simultaneous thermal gravimetric analyzer from Mettler Toledo (TGA/SDTA851e), analyses are carried out on manufactured films under nitrogen flow, and the temperature range is 30 to 800 °C at a heating rate of 20 °C/min.

#### Surface morphology

The surface morphology of the membranes was examined by Jeol JSM-6480 scanning electron microscope (SEM) operating at 30 kV. The SEM samples were coated with a layer of gold in a vacuum using an Edwards S150A sputter coater. SEM coped with Energy Dispersive X-ray Spectroscopy (SEM–EDX Model). The EDX measurements were recorded at 20 kV accelerating voltage and 21 mm working distance.

#### Mechanical properties

The mechanical characteristics of membranes were analyzed using a 100 N load cell and the Zwick Universal Test Instrument. The samples were inspected with a crosshead speed of 2 mm/min. Each sample had a minimum of five readings collected, and the average was given.

#### Porosity

Having a known area, membranes were immersed in water and weighed (W_w_) after wiping superficial water with filter paper. Keeping at room temperature for 6 h to completely dry and weigh (Wd). The porosity of the membrane (Pr) was calculated by the following equation:

$$\Pr \% = 100\,000{ } \times { }{{\left( {{\text{Ww}} - {\text{Wd}}} \right)}}/{{\text{V}}}$$

where Pr is the membrane porosity; W_w_ and W_d_ are the weights (g) of the wet and dry membranes, respectively; V = A * t where A is the membrane surface area (cm^2^), and t is the membrane thickness (cm) [29].

#### Swelling

The membranes were dried to remove any remaining water. A known weight (W_d_) was soaked in deionized water at room temperature for 24 h before being removed, wiped with tissue paper, and immediately weighed with a microbalance (W_w_). The above stages were performed daily until the equilibrium state (W_w_ constant) was reached. Finally, the membrane swelling ratio (S) was computed using the following equation [30]:

$${\text{S }}\% = \frac{{\left( {{\text{Ww}} - {\text{Wd}}} \right)}}{{{\text{Wd}}}}{ } \times { }100$$

#### Ion release

According to Yin et al. [31], the stability of anatase was investigated in a batch-release experiment. To 1 cm square membrane samples, 10 mL of deionized (DI) water was introduced and stirred at room temperature. Titanium concentrations were measured daily for 7 days using atomic absorption spectroscopy. Agilent Technologies (AA300) Knowing that the detection limit for this method is 0.04 µg/ml.

#### Antimicrobial activity

First, pour 1 ml from reference strain G+ve (*Staphylococcus aureus Enterococcus fecalis*) or G−ve (*E.coli*,* Salmonella Typhimurium*, *Enterobacter aerogenes*) on each plate. Then 15 ml tryptone soya agar (TSA) was poured into a Petri dishes. Gently, the dishes were shaken clock and anti-clock way. After solidification of the media, the membranes were put on the plate and incubated at 37 °C for 24 ± 2 h. The inhibition zone around the membranes was measured and digitally photographed [32].

#### Application of the prepared membranes

**Fig. 3:**
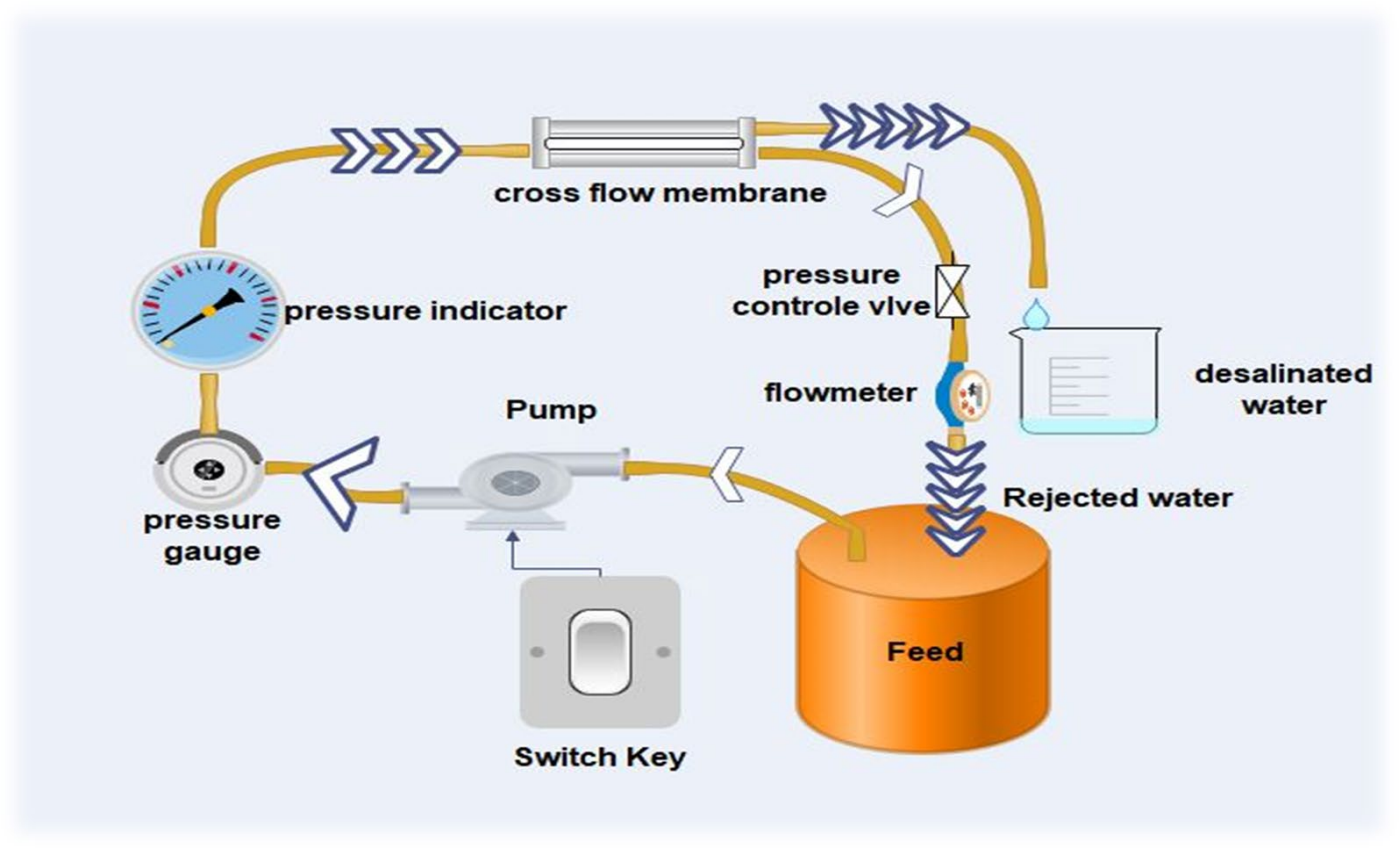
Setting up the membrane for testing

A cross-flow membrane setup system (Fig. 3) with 150 cm^2^ effective areas was used to evaluate the manufactured membrane performances. Usually, the membrane cell is covered by a circular membrane. The permeate flow was then determined at operating pressure (5:20 bar) at room temperature by forcing pure or salt water with a concentration of 98.5% across the membrane. The permeate flux *(J*) was determined by the following equation:

$$J = \frac{V}{A \times \Delta t} \times 100\%$$

where* J* (l m^−2^ h^−1^) is the permeate flux, *V* in a liter (l) represents the volume of the permeated solution, *A*(m^2^) is the effective membrane area, and $\Delta$*t* is the permeation time in an hour.

Salt rejection (*R*) was determined by the following equation:

$$R = \left( {\frac{{C_{f} - C_{p} }}{{C_{f} }}} \right) \times 100\%$$

where *C*_*f*_ (mg/ml) and* C*_*p*_ are the concentration of feed and permeate solutions, respectively, and were measured by total dissolved salt (TDS) apparatus.

## Results and discussion

### Fourier transforms infrared spectroscopy (FTIR)

**Fig. 4:**
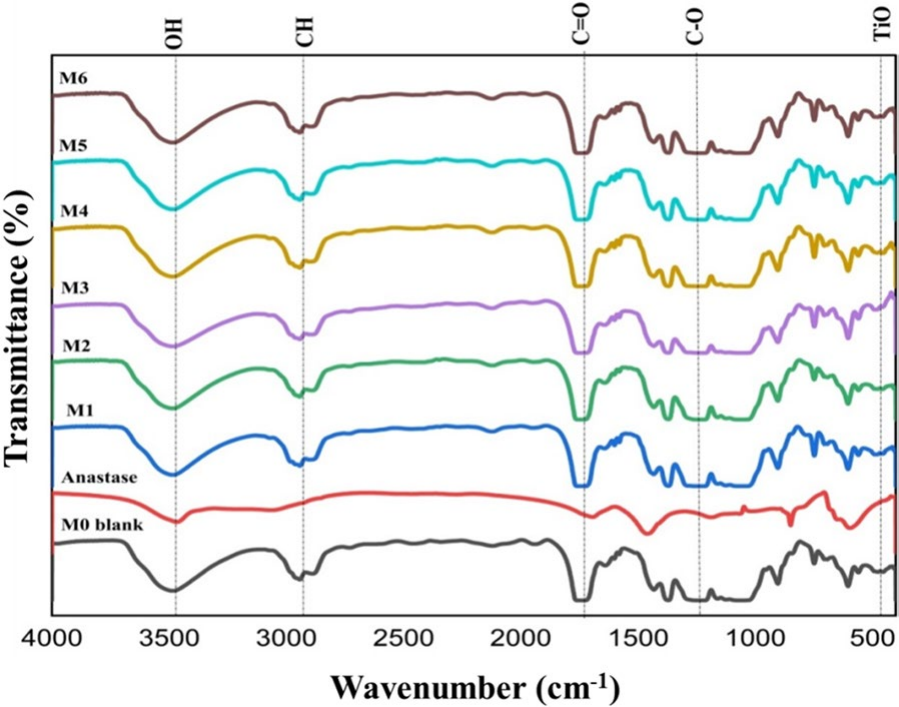
FTIR Spectra of Anatase and the prepared membranes

The FTIR spectra of CA, anatase, and their blend membranes are illustrated in Fig. 4. The blank CA membrane shows the presence of three important acetyl group vibrations at 3521 (ν_O–H_), 2965 (alkane ν_C–H_), 1771 (ν_C=O_), 1382 & 1450 (ν_–CH_ bending), 1251 (esters ν_C–O_) and 1032 cm^−1^ (anhydroglucose unit ν_C–O–C_) [33]. Peaks of anatase are visible at 3485 (*v*_O–H_), 3055, and 1697 cm^−1^(*v*_Ti–OH_). The biosynthesis of polymorphs of anatase is similar to the shield protecting *B. thuringiensis* from the harmful effects of Ultra Violet). The peaks in the blended films are the same but have varying intensities. Compared to blank CA, the OH and C=O intensities rose, demonstrating anatase incorporation. Ti–O–Ti and Ti–O–C bonds are responsible for the peak of the blended film of about 600 cm^−1^ [34].

### X-ray diffraction (XRD)

**Fig. 5:**
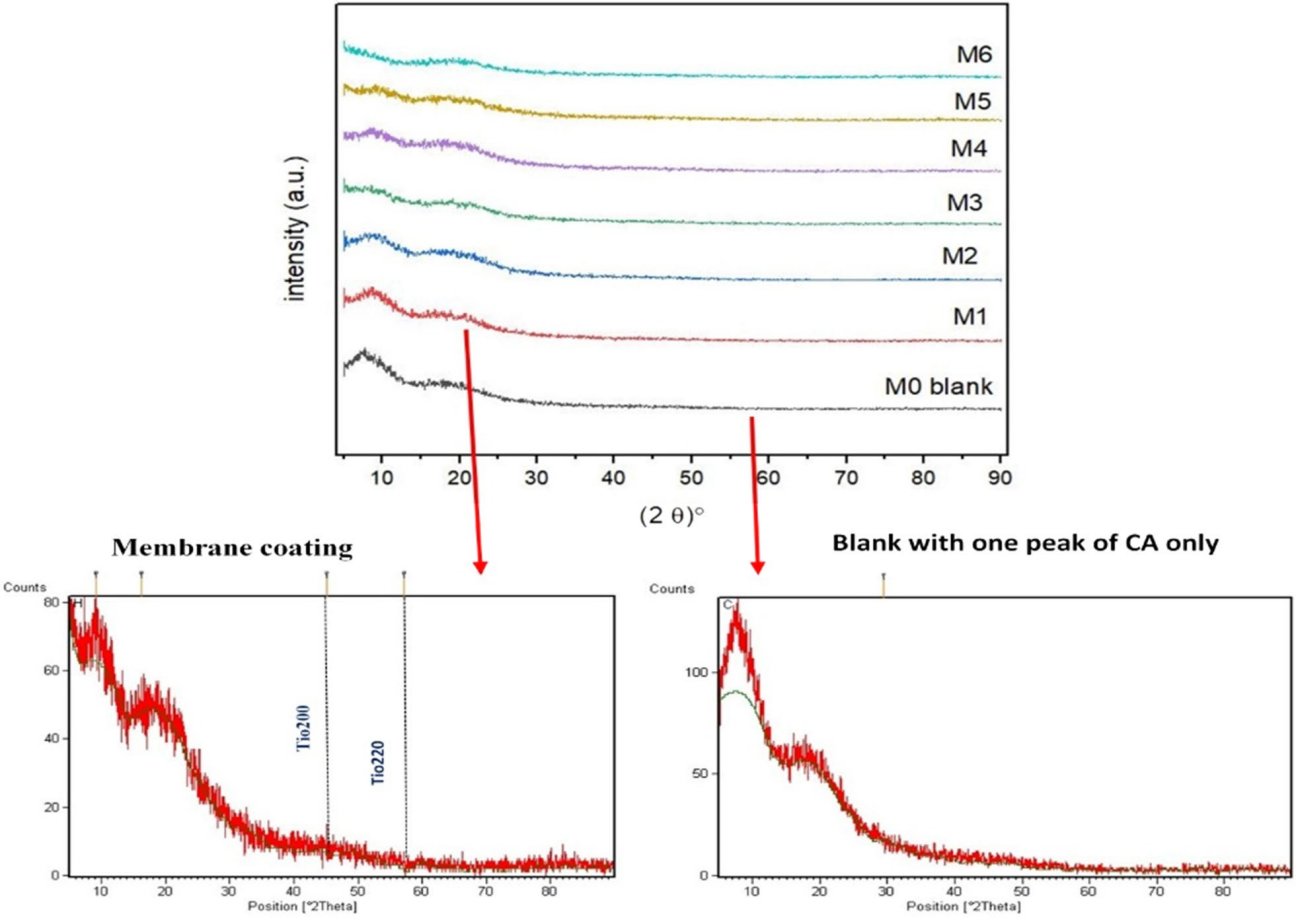
XRD patterns of the prepared membranes

The crystallinity was analyzed using X-ray diffraction (Table 1 and Fig. 5). CA displayed an amorphous signal at 2θ = 7.73 and 18.57° correlated to the plane, which showed a characteristic signal for CA [35]. The calculated crystallinity showed that the crystallinity of the blended membrane decreased compared to the blank CA, crystallinity disagrees with thermal analysis may be due to the difference in the type of H bond (intra- and inter-molecular) [25].

**Table 1:** The crystallinity index of the prepared membranes

Sample	Total area of crystalline peaks	Total area of crystalline and amorphous peaks	Crystallinity index in %
M0	537.54	1719.89	31
M1	401.68	1677.822	24
M2	381.93	1680.12	23
M3	283.89	1386.294	20
M4	255.46	1662.74	15
M5	145.09	1344.929	11
M6	43.25	1002.3	4

### Thermogravimetric analysis (TGA)

**Fig. 6:**
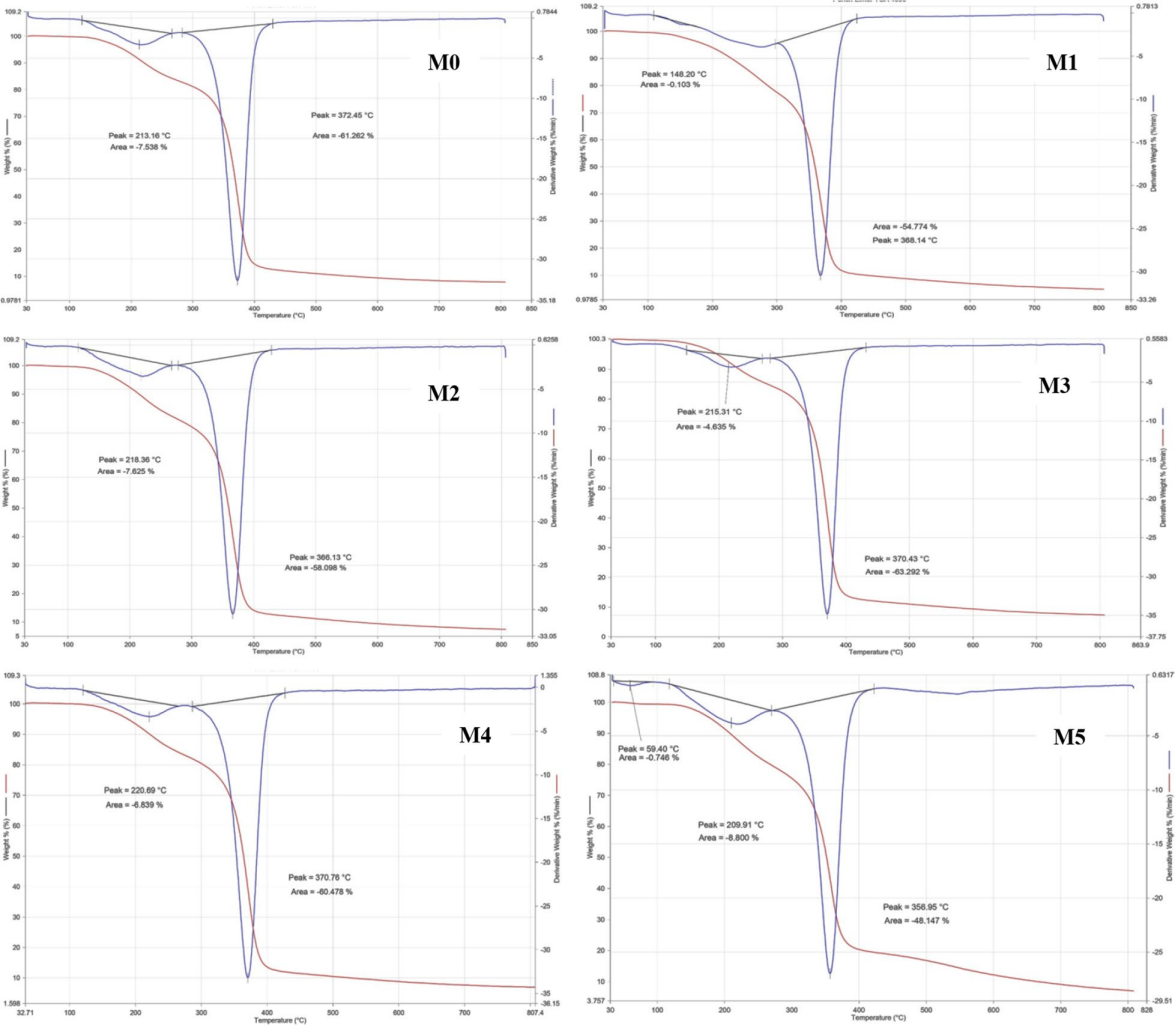
TGA of M0, M1, M2, M3, M4, and M5

The decomposition curves of M0, M1, M2, M3, M4, and M5 (Fig. 6) revealed three decomposition steps with a weight loss of 68.9, 55.1, 67.4, 68.2, 67.5, 57.6, and 66.3%, respectively, at 800 °C, which indicated a fractional existence of non-volatile contents. The first weight loss was in the range of 30–125, 30–120, 30–130, 30–100, 30–130, 35–80, and 35–80 °C with a maximum temperature of 60, 80, 45, 60, 40, 59, and 60 °C and an average weight loss of 0.2, 0.3, 0.2, 0.4, 0.3, 0.7, and 0.7%, which is most likely attributed to the loss of moisture content. The second endothermic stage is between 150–280, 120–180, 130–170, 150–250, 120–270, 130–280, and 130–270 °C, with a maximum temperature of 213, 148, 220, 215, 220, 209, and 218 °C and an average weight loss of 7.5, 0.1, 6.8, 4.6, 6.8, 8.8, and 7.6% respectively. This step is attributed to dehydroxylation reaction in combination with pyrolytic degradation. The third decomposition step was between 310–430, 290–430, 280–430, 280–430, 280–430, 270–430, and 280–430 °C with maximum temperature values at 372, 368, 370, 370, 370, 356, and 366 °C and an average weight loss of 61.2, 54.7, 60.4, 63.2, 60.4, 48.1, and 58.0%. The degradation of the remaining carbonaceous, which produces low molecular mass volatile compounds, was linked to the third decomposition process. M5 has the most significant second-step decomposition, expressed as greater thermal stability than the blank [36]. In general, the addition of anatase increased the thermal stability of CA membranes and this is shown in the results of Table 2 and Fig. 6.

**Table 2:** TGA analysis of the prepared membranes

Sample	Thermal degradation step	Weight loss (%)	Residual %	Tonset (◦C)	Tpeak (◦C)	Tendset (°C)
M0	Stage 1	7.5	92.5	120.0	213.2	270.0
	Stage 2	61.2	38.8	285.0	372.4	430.0
M1	Stage 1	4.6	95.4	147.8	215.3	268.8
	Stage 2	63.2	36.8	281.7	370.4	430.1
M2	Stage 1	6.8	93.2	118.2	220.6	263.1
	Stage 2	60.4	39.6	285.5	370.7	427.6
M3	Stage 1	0.1	99.9	108.4	148.2	182.5
	Stage 2	54.7	45.3	298.0	368.14	425.1
M4	Stage 1	0.7	99.3	34.5	59.4	94.7
	Stage 2	8.8	91.2	114.1	209.9	267.5
	Stage 3	48.1	51.9	267.5	356.9	422.4
M5	Stage 1	0.7	99.3	4.0	59.4	91.2
	Stage 2	8.8	91.2	115.2	209.9	268.4
	Stage 3	48.1	51.9	168.4	356.9	422.8

### Surface morphology

**Fig. 7:**
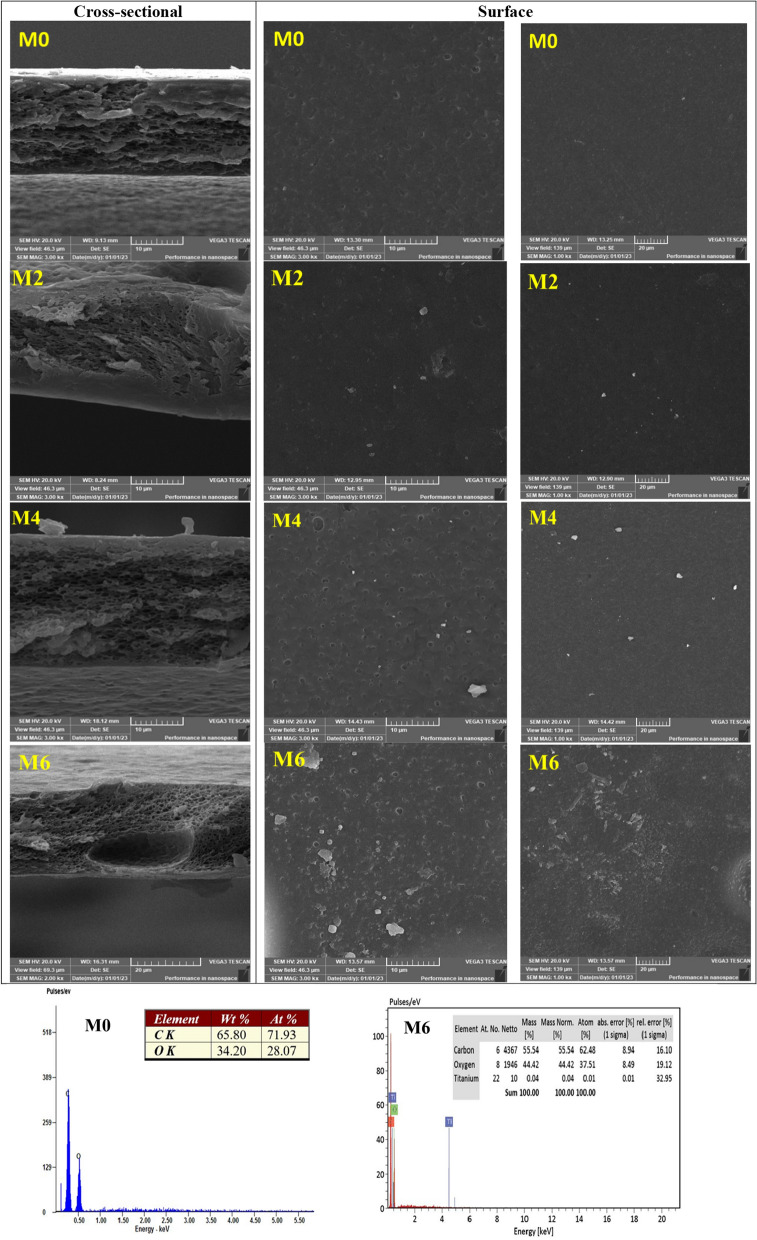
Cross-sectional and surface SEM images of M0, M2, M4, and M6 membranes with the EDX analysis of M0 and M6

Figure 7 shows the cross-sectional and surface SEM images of the prepared membranes to recognize porosity scattering in membranes. SEM of the CA membrane was observed with a top-dense skin layer. It was evident from the SEM that the existence of nano-sheets, which has an impact on the structure of membrane-forming in the phase inversion process, is primarily responsible for the variable pore size distribution of the created membranes. By adding anatase, the membrane’s porosity decreased; this phenomenon’s appearance may be attributed to the higher chance of agglomeration for anatase. Overall, the top surfaces of all the prepared membranes revealed are dense structures. Meanwhile in the cross sections, the top surface was supported in the intermediate by a spongy-like structure, followed by a macrovoids/finger-like structure, at the end by the last bottom surface layers shown in EDX investigations. The revealed peak at about 3 keV corresponds to Titanium (Fig. 7) [37].

### Mechanical properties

**Fig. 8:**
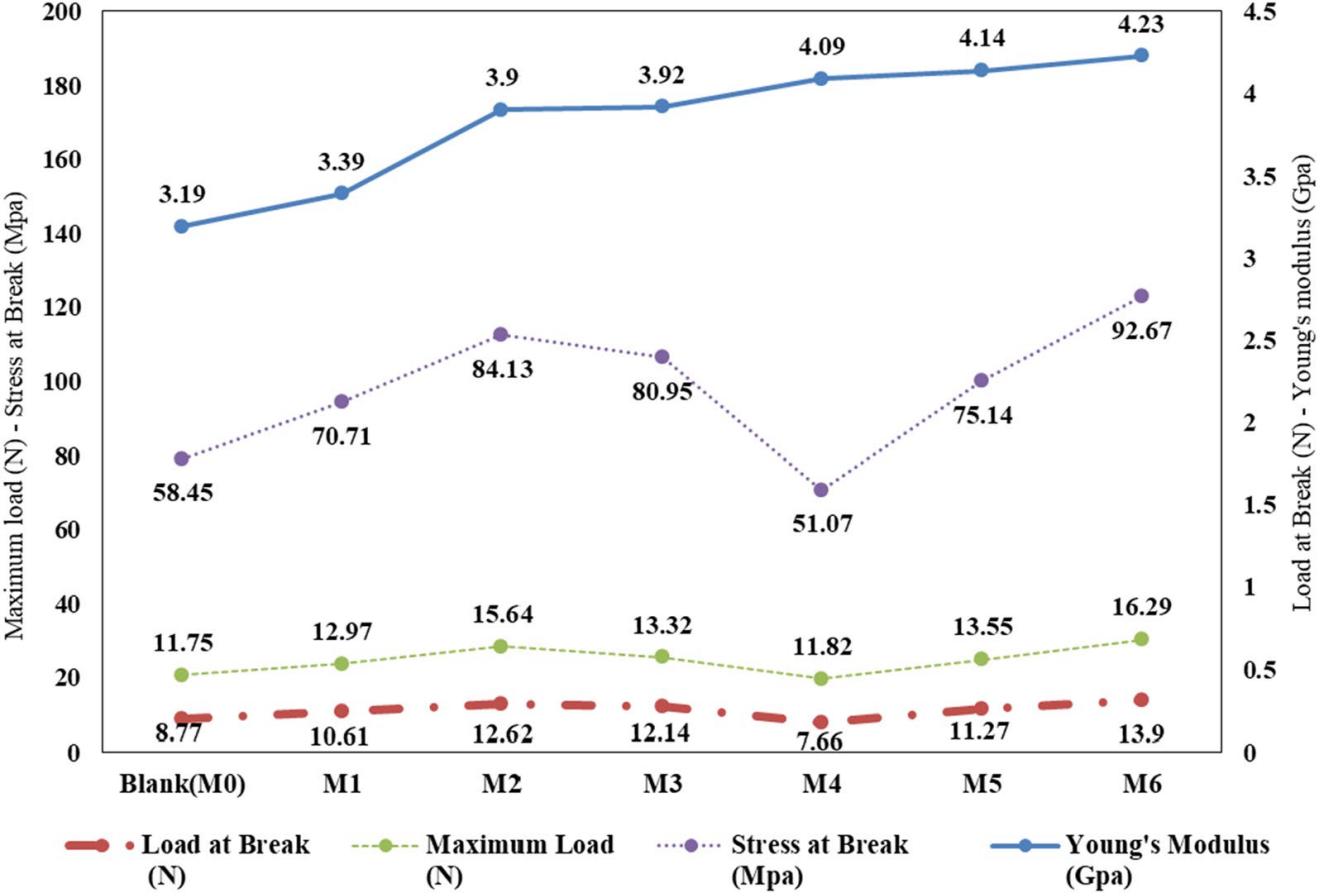
Mechanical properties of the prepared membranes

Figure 8 depicts the mechanical properties of the prepared membranes. Anatase was added to improve the membrane’s properties, and Young’s modulus increased steadily as anatase content increased. The burden at break initially increased with the addition of anatase. It peaked when the anatase ratio was between two and three percent and began to fall when more anatase was added. Membrane maximum load measurements revealed a similar fluctuating pattern. The maximal load increased with anatase addition, peaking at 2% then decreasing with the anatase ratio rise. Furthermore, stress levels throughout the break followed a similar trend, peaking at 2% anatase. These patterns could be caused by the addition of anatase, which increases the viscosity of CA solutions [29].

Finally, the composite membranes containing 3% anatase had the best mechanical characteristics. Maximum load increased from 11.75 N of pure CA membrane to 15.64 MPa of anatase-loaded CA membrane. The ideal membranes’ stress at break might reach 84.13 MPa, compared to 58.45 MPa for the CA membrane.

### Porosity determination & swelling ratio of membrane

**Fig. 9:**
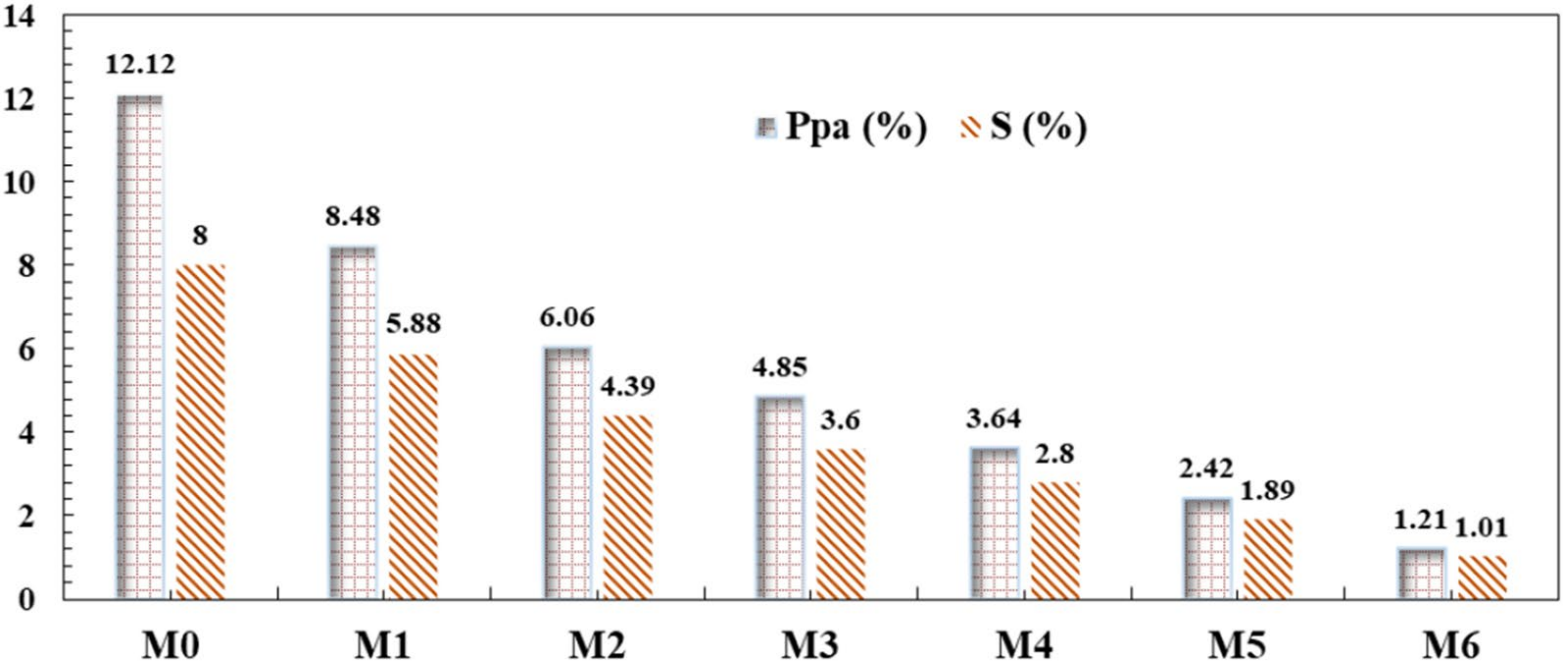
Swelling and porosity of the prepared membranes

The effect of anatase on the CA membranes’ porosity and swelling are described in Fig. 9. Both porosity and swelling were decreased with increasing anatasecontent in the membrane. The porosity of membranes decreased from 12.12% of anatase-free CA to 1.21% of 0.6% of anatase-loaded CA membranes. A similar variation trend was also exhibited with swelling that dropped from 8 to 1.01%, increasing anatase from 0 to 0.6%. These trends may be explained as follows; due to the hydrophilic nature of CA, it contains fewer active groups that can form hydrogen bonds with water molecules. By adding anatase, the matrix will be bounded by crosslinking, limiting water penetration inside the matrix.

### Stability of anatase-containing CA membranes

The anatase-containing CA membrane’s stability was studied for 7 days to evaluate the release of TiNPs from membranes. The sample with dimensions of (L × W) 1 × 1 cm was soaked in a tube with 10 ml deionized (DI) water and shacked at room temperature. However, anatase is an inert and safe material and has been used in many applications for decades because of its photocatalyst and non-toxicity property and has attractive for water treatment. The release rate of TiNPs from the CA-loaded anatase membranes would control the duration of the effectiveness of membranes; consequently, the CA-loaded anatase was more stable for industrial applications in desalination. We studied the possibility of TiNPs release from the prepared membranes during a day, 3 days, a week, and 2 weeks, and it was inferred that there are no traces of titanium in the ionized water.

### Evaluating the membrane performance

**Fig. 10:**
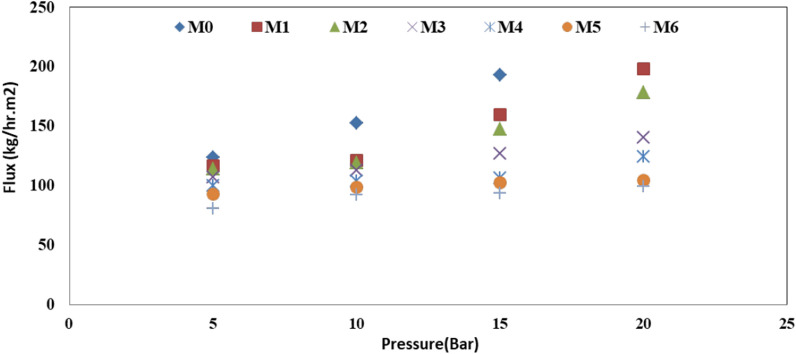
Water flux at operating condition 10 bar and feed concentration 5 g/l NaCl

**Fig. 11:**
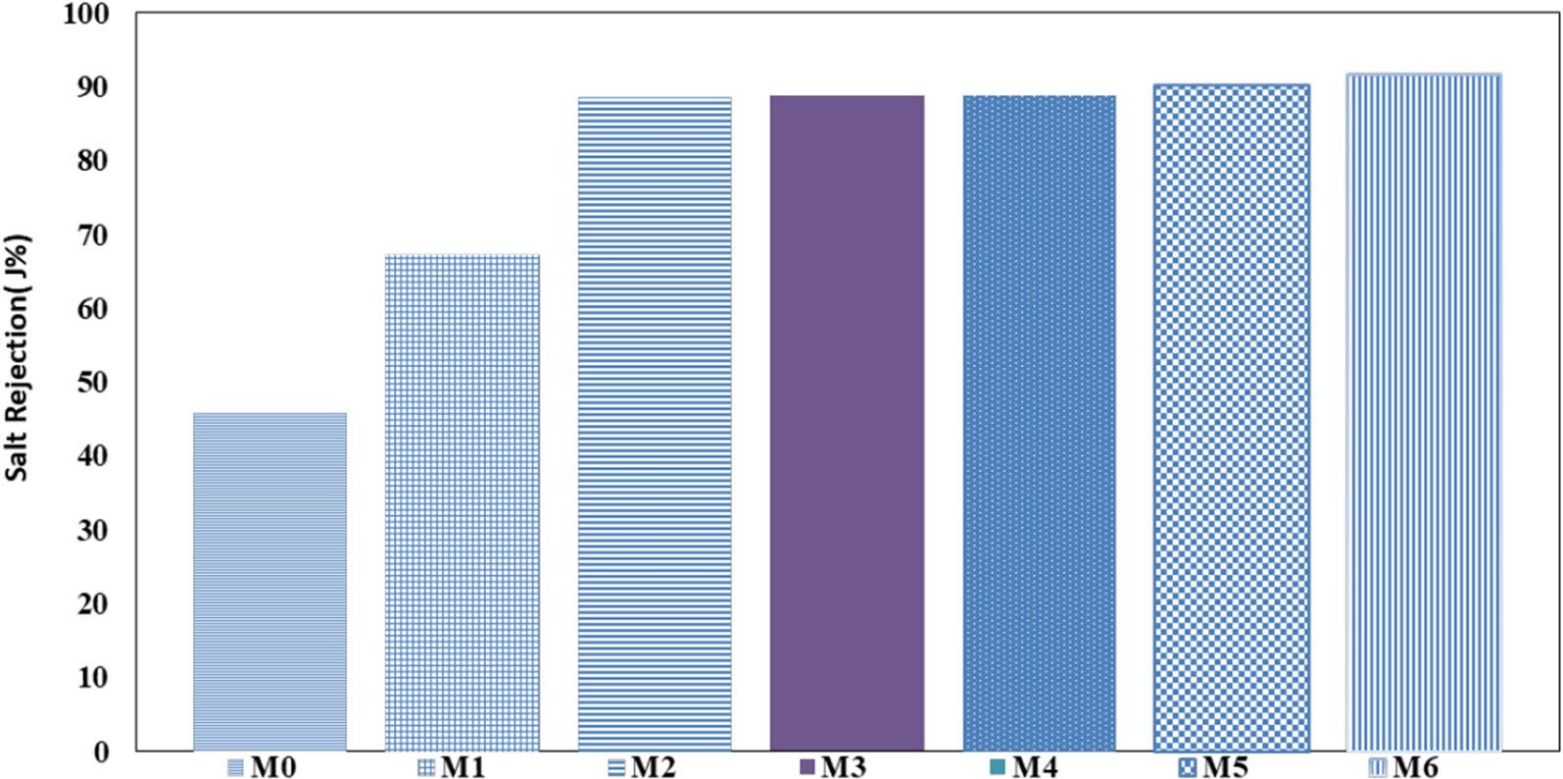
Effect of membrane type on salt rejection at operating condition 10 bar and feed concentration 5 g/l NaCl

The membrane performance was evaluated in this part by measuring permeate flux (*J*) and salt rejection (*R*) of the prepared CA/anatase membranes, as displayed in Figs. 10 and 11. It was clear from Fig. 10 that the permeate flux is increased with the increasing operating pressure for all tested membranes following Darcy’s law [38]. It was evident from Fig. 10 that the permeate flux of prepared CA/anatase membranes (M1:M6) was decreased with the addition of nanomaterial following this sequence; M0> M1> M2> M3> M4> M5> M6. With the addition of nanomaterial, the permeate flux of the prepared CA/anatase membranes is changed significantly, decreasing from 150: 90 kg/m^2^h with M0: M6 at operating condition 10 bar and feed concentration 5 g/l NaCl. This effect could be attributed to the change in prepared membrane morphology with the addition of the nanomaterial. Meanwhile, high anatase content shows lower porosity than a blank membrane; this result is proved by the porosity and swelling results which have the same impact; the anatase content is uniformly embedded in the polymer matrix resulting in the decrease of membrane flux. This result could be attributable to CA’s hydrophilic nature; it contains fewer active groups that can form hydrogen bonds with water molecules. By adding nanoparticle, the polymer matrix will be bounded by crosslinking, which decrease the permeate flux inside the prepared CA/anatase membrane.

It was apparent from Fig. 11 that the salt rejection of prepared CA/anatase membranes (M1:M6) was improved with the addition of nanomaterial following this sequence; M6> M5> M4> M3> M2> M1> M0. With the addition of nanomaterial, the salt rejection of prepared CA/anatase membranes is changed significantly, increasing from 46 to 92% with M0: M6 at operating condition 10 bar and feed concentration 5 g/l NaCl. This result can be ascribed to the modification of prepared membrane morphology by adding the nanomaterial. In the meantime, high anatase content shows lower porosity than blank membrane; the anatase content is uniformly embedded in the polymer matrix resulting in increased membrane salt rejection [23].

### Antimicrobial studies

**Fig. 12:**
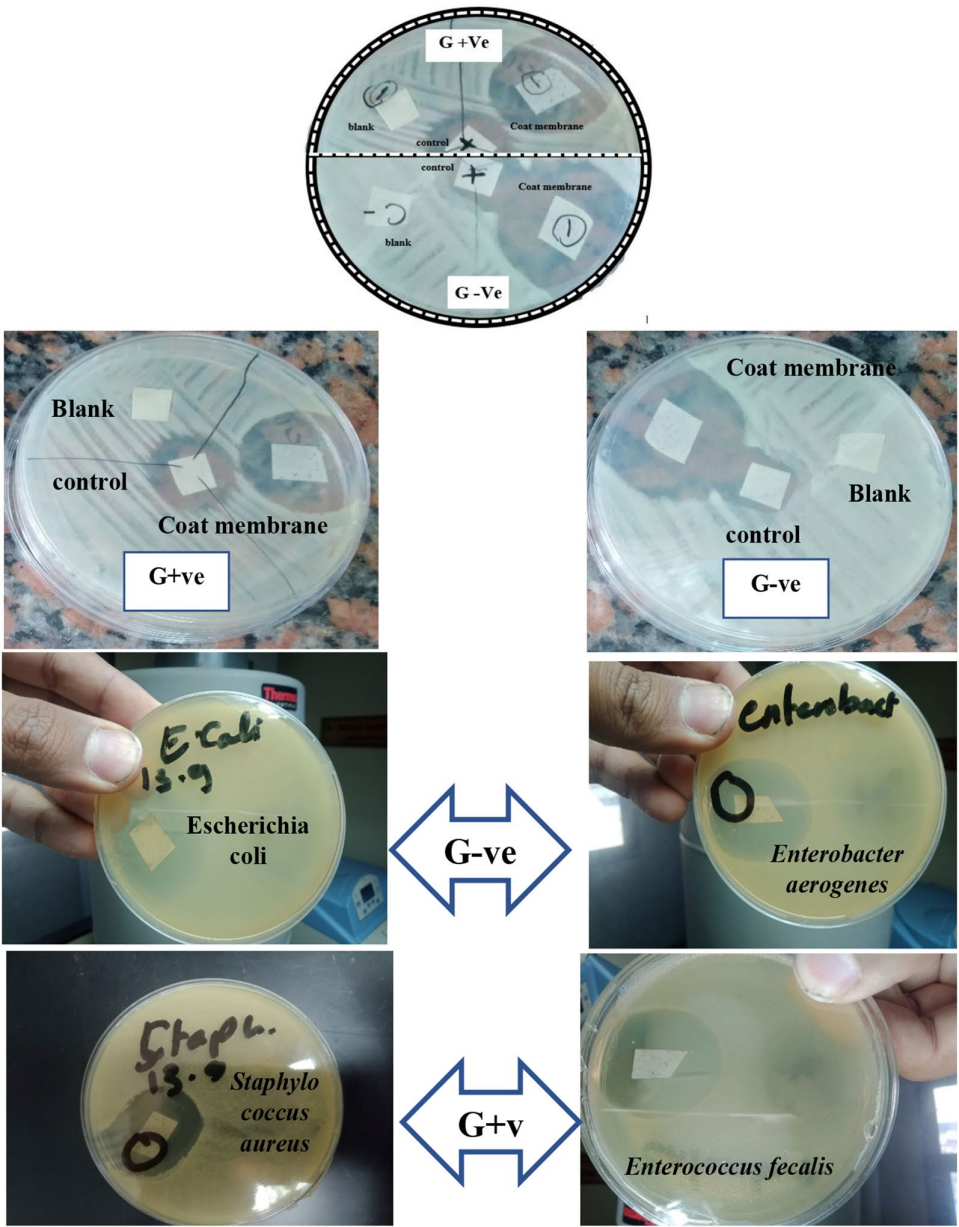
Antimicrobial assay of CA and CA/anatase membranes

The antibacterial activity of the prepared membranes (CA and CA/anatase) against various Gram-positive and Gram-negative bacterial strains was tested (Table 3). Overall, there is no inhibition zone in the blank CA membrane. The composite membrane (CA and CA/anatase), on the other hand, exhibited a broad inhibition zone against Gram-positive and Gram-negative pathogens. For example, the composite membrane showed an inhibition zone with a diameter of 25 and 20 mm against *Escherichia coli and Salmonella* *Typhimurium* (Gram-negative bacterial strains), respectively, while the diameter of the inhibition zone was 25 and 15 mm in the case of *Staphylococcus aureus* and *Enterococcus fecalis* (Gram-positive bacterial strains), respectively (Fig. 12). These findings are consistent with those of the APHA [39].

**Table 3:** Inhibition zone of CA and CA/anatasemembrane against bacterial strains after incubation at 37° C for 24 h

Bacteria Strains		CA	CA/anatase
G−Ve	*E.coli*	No zone	15
	*Salmonella* *Typhimurium*	No zone	20
	*Enterobacter aerogenes*	No zone	15
G+Ve	*Staphylococcus aureus*	No zone	25
	*Enterococcus fecalis*	No zone	15

### Comparison with previous works

After adding TiO_2_ NPs to TiO_2_/Ph-CA membrane, it raised its thermal stability and lowered the water absorption and swelling ratios when compared with the original and functional reference, these results are consistent with Khalifa et al. [40]. Also, the water flowing through the prepared membrane in the current study is acceptable when compared with the flow rate mentioned in previous studies [41, 42] using (Ti3C2T×(MXene)/C) and (CA/SiO_2_/TiO_2_) membranes.

SEM results showed some agglomerations on the skin layer of the membranes where the lower the concentration meaning the better its porosity. This is like nickel ferrite (NiFe_2_O_4_) and titanium dioxide (TiO_2_) nanoparticles on CH_4_/CO_2_ gas permeability properties of cellulose acetate-based mixed matrix membranes [43].

The composite membrane showed strong antibacterial features shown when using Gram- positive and negative strains. The results showed clear zones of inhibition and this corresponds to electrospun PVDF/CA nanofiber membranes with different concentrations of TiO_2_ that showed antibacterial activity against both Gram- negative and positive strains [44].

## Conclusion

Cellulose acetate/anatase (different ratios) hybrid membranes were successfully prepared via Loeb–Sourirjan wet phase inversion process.

The membrane properties were affected by the ratio of loading anatase. The main conclusions can be listed as follows:

- The high content of anatase decreased the porosity and swelling of the membrane.
- The permeate flux rose with increasing operating pressure for the membrane without anatase. In comparison, the permeate flux decreased with the addition of anatase and decreased with increasing anatase ratio.
- The permeability flux of the CA/anatase membranes altered considerably with the addition of anatase, ranging from 150 to 90 kg/m^2^ for M0 and M6, respectively, with 10 bar pressure and 5 g/l NaCl feed concentration.
- The addition of anatase enhanced the salt rejection of the membranes and increased with increasing anatase ratio; 46% for the membrane without anatase and 92% for high-loading anatase.
- Loading of anatase-created antimicrobial efficiency for cellulose acetate membrane against Gram-positive and Gram-negative bacteria.

In general, loading anatase to cellulose acetate membrane is a promising and straightforward method to produce anti-fouling and anti-bacterial membranes.

## Data Availability

The datasets used and/or analyzed during the current study available from the corresponding author on reasonable request.

## Abbreviations

CTA: Cellulose tri-acetate membrane
TSA: Tryptone soya agar
TFN: Thin film nanocomposite
NPs: Nanoparticles
RP: Reverse osmosis
CA: Cellulose acetate
CMC: Carboxymethyl cellulose
L–S: Lob–Surian
PPA: Membrane porosity
Wd or Wdry: Dry membrane weight
Ww or Wwet: Membranes Weight
S%: Swelling ratio
TGA: Thermogravimetric analysis
SEM: Scanning electron microscopy
FTIR: Fourier transform infrared
N: Newton
GPa: Gega Pascal
MPa: Mega Pascal
*E.coli*: *Escherichia coli*
FM: Filter membrane
Ti: Titanium
MCM: Million cubic meters
Pr: Membrane porosity
g: Gram
h: Hour
nm: Nano meter, A = X * L = 2.5 * 5.5, T = 0.02 mm,V = 2.5 * 5.5 * 0.2 = 2.75 cm^3^
